# Dilatation mitrale percutanée des rétrécissements mitraux en hypertention pulmonaire importante au cours de la grossesse

**DOI:** 10.11604/pamj.2021.40.265.11297

**Published:** 2021-12-30

**Authors:** Fatima Azzahra Benmessaoud, Nessema Bendagha, Aida Soufiani, Lassana Konaté, Nadia Fellat, Rajae Benani, Naima El Haitam, Roukia Fellat

**Affiliations:** 1 Service de Cardiologie B, CHU Ibn Sina, Rabat, Maroc,; 2 Service de Cardiologie A, CHU Ibn Sina, Rabat, Maroc

**Keywords:** Grossesse, rétrécissement mitral, dilatation mitrale percutanée, Pregnancy, mitral stenosis, percutaneous dilation of mitral stenosis

## Abstract

Le rétrécissement mitral (RM) rhumatismal est la valvulopathie organique la plus fréquente dans les pays en voie de développement. La grossesse constitue l´une des circonstances de décompensation de cette valvulopathie. Nous rapportons notre expérience dans la dilatation mitrale percutanée des rétrécissements mitraux en hypertension pulmonaire importante au cours de la grossesse. La commissurotomie mitrale percutanée (CMP) a été réalisée chez deux cent vingt-trois parturientes entre janvier 2009 et décembre 2015. Quarante trois soit 19% de ces patientes avaient une hypertension pulmonaire importante (PAPS > 70 mmHg). Toutes les parturientes de notre série sont porteuses d´un RM très serré symptomatique malgré un traitement médical bien conduit. La CMP a été un succès chez l´ensemble des patientes de l´étude, toutes les patientes se sont améliorées sur le plan clinique. L´insuffisance mitrale a progressé d´un grade chez deux patientes. Une patiente a présenté une tamponnade avec une évolution favorable après ponction péricardique. Aucun avortement n´est survenu après procédure et deux accouchements prématurés ont été rapportés. La prise en charge d´un RM serré durant la grossesse doit être multidisciplinaire et faire intervenir le gynécologue-obstétricien, l´anesthésiste et le cardiologue. La CMP constitue actuellement le traitement de référence du RM au cours de la grossesse.

## Abstract (en)

Rheumatic mitral stenosis is the most common organic valvular heart disease in developing countries. These patients are at risk of decompensation during pregnancy. We here describe our experience with percutaneous dilation of mitral stenosis in patients with severe pulmonary hypertension during pregnancy. Percutaneous balloon mitral valve commissurotomy was performed in two hundred and twenty-three pregnant women between January 2009 and December 2015. Forty-three (19%) of these patients had severe pulmonary hypertension (SPAP > 70 mmHg). All pregnant women in our study had very severe symptomatic rheumatic mitral stenosis despite well-performed medical treatment. All patients had clinical improvement after percutaneous balloon mitral valve commissurotomy. The severity of mitral insufficiency progressed by one grade in two patients. One patient had tamponade with favorable outcome after a pericardial puncture. No abortion occurred after the procedure and two preterm deliveries were reported. Patients with severe rheumatic mitral stenosis during pregnancy should receive multidisciplinary care involving an obstetrician, anesthetist and cardiologist. Percutaneous balloon mitral valve commissurotomy is currently the standard treatment for rheumatic mitral stenosis during pregnancy.

## Introduction

Le rétrécissement mitral (RM) rhumatismal est la valvulopathie organique la plus fréquente dans les pays en voie de développement. Selon *Euro Heart survey*, étude prospective menée sur 5 000 patientes, dans 85,4% des cas l´étiologie de cette valvulopathie est rhumatismale [1]. La grossesse constitue l´une des circonstances de décompensation de cette valvulopathie. En effet, entre la 20^e^ et la 24^e^ semaine d´aménorrhée, le débit cardiaque augmente de 40 à 50% de façon à satisfaire la circulation fœto-placentaire [2], constituant ainsi une source de décompensation du rétrécissement mitral serré au cours de la grossesse. La prise en charge chirurgicale des cardiopathies valvulaires au cours de la grossesse mettait en jeu le pronostic vital maternel et foetal. Aussi, la commissurotomie mitrale percutanée (CMP) durant la grossesse constitue, une alternative séduisante au traitement chirurgical.

## Méthodes

La commissurotomie mitrale percutanée (CMP) a été réalisée chez deux cent vingt-trois parturientes entre janvier 2009 et décembre 2015. Quarante trois soit 19% de ces patientes avaient une hypertension pulmonaire importante (PAPS > 70 mmHg). Toutes les parturientes de notre série sont porteuses d´un RM très serré symptomatique malgré un traitement médical bien conduit.

**Les critères d´inclusion étaient les suivants:** RM serré avec une surface mitrale 1,5 cm^2^; classe NYHA ≥ 2; absence d´insuffisance mitrale (IM) sévère ( ≥ à un grade III); absence de thrombus de l´OG ou de l´auricule gauche; durée de gestation > 20 SA; pressions artérielles pulmonaires systoliques > 70 mmHg; l´âge moyen de nos patientes est de 30 ans, 85% de nos patientes ont des antécédents d´angine à répétition et 26% des antécédents de rhumatisme articulaire aigu; à l´admission 40% de nos patientes avaient un œdème aigu pulmonaire et 26 % avait des signes d´insuffisance cardiaque droite. Seulement 40% des patientes étaient en rythme sinusal.

**Evaluation échocardiographique:** une écho cardiographie Doppler a été réalisée avant et après la procédure chez toutes les patientes. La sévérité du rétrécissement mitral est évaluée par planimétrie associée à une analyse minutieuse de l´appareil valvulaire et sous valvulaire. Les PAPS ont été évaluées sur le flux de l´insuffisance tricuspide. Une échocardiographie transœsophagienne a été effectuée systématiquement avant la dilatation mitrale percutanée.

**Commissurotomie mitrale percutanée:** pour limiter l´irradiation durant la procédure, nous avons eu recours à une protection de l´abdomen maternel par une coquille de plomb. La scopie n´a été utilisée que si nécessaire. Un cathéter 6F pigtail a été placé au niveau de la racine de l´aorte. La CMP a été réalisée selon la technique d´Inoué, une ponction transeptale de l´oreillette gauche a été effectuée grâce à l´aiguille de Brockenbrough introduite via la veine fémorale droite. L´héparine a été administrée après la ponction transeptale. L´inflation du ballon d´Inoué a été faite progressivement jusqu´à un diamètre permettant la disparition de l´empreinte mitrale en scopie, en incidence oblique antérieure droite à 30°. La pression moyenne de l´oreillette gauche (OG) a été mesurée avant et après la procédure. La CMP est considérée comme réussie si la surface a augmenté de 50% par rapport à la surface de départ avec une surface mitrale post dilatation > 1,5 cm^2^ en l´absence d´IM significative (supérieure ou égale à un grade III).

## Résultats

La CMP a été un succès chez l´ensemble des patientes de l´étude, toutes les patientes se sont améliorées sur le plan clinique. La pression de l´OG abaissée de 34,4±5,4 à 14,3±6 mm Hg. Le gradient moyen transmitral est passé de 25,1±7,1 à 8,5±4,1 mmHg. Le temps de scopie moyen était de 4±3,1 minutes. Les PAPS sont passées de 70±40 à 30±15mmHg. La surface mitrale a augmenté de 0,7 ± 0,2 à 1,5±0,5 cm^2^. L´insuffisance mitrale a progressé d´un grade chez deux patientes. Une patiente a présenté une tamponnade avec une évolution favorable après ponction péricardique. Aucun avortement n´est survenu après procédure et deux accouchements prématurés ont été rapportés.

## Discussion

En cas de rétrécissement mitral serré, même bien toléré antérieurement, le risque de décompensation est élevé durant la grossesse. La plupart des patientes en classe fonctionnelle NYHA I ou II avant la grossesse voient ainsi leurs symptômes s´aggraver [3]. Les modifications physiologiques de l´hémodynamique lors de la grossesse consistent en une augmentation de la volémie, une tachycardie, une diminution des résistances artérielles systémiques et une augmentation du débit cardiaque [4]. L´augmentation du débit cardiaque est le principal facteur susceptible de décompenser le rétrécissement mitral.

Comme le gradient à travers la valve est proportionnel au débit cardiaque et que ce dernier augmente pendant la deuxième moitié de la grossesse, le gradient transvalvulaire s´élève d´environ 25% [5]. D'autre part, la tachycardie qui accompagne l'augmentation du débit raccourcit dangereusement la diastole et diminue ainsi le débit antérograde [5]. Par conséquent cet état d´hypervolémie associé au rétrécissement mitral serré entraine une augmentation des pressions de l´OG et des pressions capillaires pulmonaires constituant un lit pour les complications materno-fœtales [3]. L´œdème aigu pulmonaire et les arythmies représentent les complications maternelles les plus fréquentes et elles dépendent de la gravité de la sténose mitrale ainsi que de la classe fonctionnelle NYHA [6,7], d´ailleurs près de la moitié de nos patientes étaient en OAP à l´admission versus 7% chez les parturientes ayant une sténose mitrale et une HTP modérée , ce qui conforte la corrélation entre la gravité des symptômes et l´importance de l´HTP .

En ce qui concerne les complications fœtales, la prématurité est la complication fœtale la plus observée [6]. La mortalité maternelle passe de moins de 1% pour les stades I-II à 7% pour les stades III et IV de la NYHA, la mortalité fœtale est de 30% pour le stade IV de la NYHA et la période la plus critique se situe lors du travail et de la délivrance [8]. L´échographie est nécessaire pour évaluer la sévérité de la sténose (significative si inférieure à 1.5cm^2^ en planimétrie). La mesure du gradient mitral moyen et de la pression artérielle (PA) pulmonaire systolique sont essentiels pour évaluer l´évolution de la tolérance de la sténose mitrale durant la grossesse [5]. L´échocardiographie permet également d´évaluer l´anatomie valvulaire et la régurgitation mitrale, ce qui est particulièrement important pour évaluer la faisabilité d´une commissurotomie mitrale percutanée. Une échocardiographie transœsophagienne doit être systématiquement réalisée avant une commissurotomie mitrale percutanée afin d´éliminer un thrombus de l´oreillette gauche.

Idéalement, pour éviter la survenue des complications, le RM doit être diagnostiqué et traité avant la grossesse, mais 40 % des rétrécissements mitraux sont découverts au cours de la grossesse. Devant l´installation des symptômes [9], il faut instaurer en premier un traitement médical à base de béta-bloquant puis secondairement des petites doses de diurétique peuvent être associées en cas de persistance des symptômes. Le traitement anticoagulant n´est indiqué qu´en cas de fibrillation auriculaire ou d´antécédent embolique [10]. Lorsqu´une femme reste symptomatique malgré un traitement médical bien conduit ou si elle présente des pressions artérielles pulmonaires systoliques > 55mmHg, le risque de complications fœtales, et surtout maternelles, lors de l´accouchement justifie une intervention sur la valve mitrale durant la grossesse. La méthode de choix reste la dilatation mitrale percutanée qui doit être réalisée dans un centre de référence par une équipe entrainée, entourée de précautions particulières, et proposée à partir de la 20^e^ SA [10]. Ainsi, lors de la procédure la réduction de l´irradiation doit être maximale et le port d´un tablier en plomb au niveau de l´abdomen contribue à la protection fœtale. Selon une étude menée par bennis *et al*. La commissurotomie mitrale percutanée donne de bons résultats au cours de la grossesse avec un taux de réussite de la procédure de 98% sur les 70 patientes de la série [11], ce qui est comparable à notre série puisque nous avons eu un taux de réussite de 98% et un taux de complications materno-fœtales de 0,9% .

## Conclusion

La prise en charge d´un RM serré durant la grossesse doit être multidisciplinaire et faire intervenir le gynécologue- obstétricien, l´anesthésiste et le cardiologue. La CMP constitue actuellement le traitement de référence du RM au cours de la grossesse [12,13]. La CMP, reste donc une technique efficace, sure et sans danger pour la parturiente et pour le nouveau-né.

### Etat des connaissances sur le sujet

- *La grossesse constitue une occasion de décompensation du rétrécissement mitral serré;*
- *La prise en charge chirurgicale est abandonnée car elle engage aussi bien le pronostic maternel que fœtal;*
- *La dilatation mitrale percutanée au cours de la grossesse constitue une alternative séduisante à la prise en charge chirurgicale*.

### Contribution de notre étude à la connaissance

- *Notre étude prouve que lorsque la dilation mitrale percutanée au cours de la grossesse est réalisée dans un centre de référence et dans des conditions optimales avec respect des indications cela permet une prise en charge optimale des sténoses mitrales*.
